# Structure and release function of fragrance glands

**DOI:** 10.1093/hr/uhaf031

**Published:** 2025-02-01

**Authors:** Yunyi Chen, Ziying Jiang, Sihui Wu, Bixuan Cheng, Lijun Zhou, Tinghan Liu, Chao Yu

**Affiliations:** State Key Laboratory of Efficient Production of Forest Resources, Beijing Key Laboratory of Ornamental Plants Germplasm Innovation and Molecular Breeding, Beijing Laboratory of Urban and Rural Ecological Environment, National Engineering Research Center for Floriculture, School of Landscape Architecture, Beijing Forestry University, No. 35 Qinghua East Street, Haidian District, Beijing 100083, China; State Key Laboratory of Efficient Production of Forest Resources, Beijing Key Laboratory of Ornamental Plants Germplasm Innovation and Molecular Breeding, Beijing Laboratory of Urban and Rural Ecological Environment, National Engineering Research Center for Floriculture, School of Landscape Architecture, Beijing Forestry University, No. 35 Qinghua East Street, Haidian District, Beijing 100083, China; State Key Laboratory of Efficient Production of Forest Resources, Beijing Key Laboratory of Ornamental Plants Germplasm Innovation and Molecular Breeding, Beijing Laboratory of Urban and Rural Ecological Environment, National Engineering Research Center for Floriculture, School of Landscape Architecture, Beijing Forestry University, No. 35 Qinghua East Street, Haidian District, Beijing 100083, China; State Key Laboratory of Efficient Production of Forest Resources, Beijing Key Laboratory of Ornamental Plants Germplasm Innovation and Molecular Breeding, Beijing Laboratory of Urban and Rural Ecological Environment, National Engineering Research Center for Floriculture, School of Landscape Architecture, Beijing Forestry University, No. 35 Qinghua East Street, Haidian District, Beijing 100083, China; State Key Laboratory of Efficient Production of Forest Resources, Beijing Key Laboratory of Ornamental Plants Germplasm Innovation and Molecular Breeding, Beijing Laboratory of Urban and Rural Ecological Environment, National Engineering Research Center for Floriculture, School of Landscape Architecture, Beijing Forestry University, No. 35 Qinghua East Street, Haidian District, Beijing 100083, China; State Key Laboratory of Efficient Production of Forest Resources, Beijing Key Laboratory of Ornamental Plants Germplasm Innovation and Molecular Breeding, Beijing Laboratory of Urban and Rural Ecological Environment, National Engineering Research Center for Floriculture, School of Landscape Architecture, Beijing Forestry University, No. 35 Qinghua East Street, Haidian District, Beijing 100083, China; State Key Laboratory of Efficient Production of Forest Resources, Beijing Key Laboratory of Ornamental Plants Germplasm Innovation and Molecular Breeding, Beijing Laboratory of Urban and Rural Ecological Environment, National Engineering Research Center for Floriculture, School of Landscape Architecture, Beijing Forestry University, No. 35 Qinghua East Street, Haidian District, Beijing 100083, China

## Abstract

Volatile compounds serve physiological, signaling, and defensive purposes in plants and have beneficial effects on the growth, reproduction, resistance, and yield of horticultural plants. They are released through fragrance glands and become gasses by passing through the plasma membrane, cell walls that contain water, and cuticle. Transporter proteins facilitate their transport and reduce the resistance of these barriers. They also regulate the rate of release and concentration of volatiles inside and outside of the membrane. However, there has been no summary of the structure and function of the fragrance glands of horticultural plants, as well as an introduction to the latest research progress on the mechanism of the transport of volatiles. This review focuses on the structure and function of the release of aromas in horticultural plants and explores the mechanism of the release of volatiles through a transporter model. Additionally, it considers the factors that affect their release and ecological functions and suggests directions for future research.

## Introduction

Horticultural plants, including fruit trees, vegetables, ornamentals, and aromatic and medicinal plants, have been cultivated by humans for a long time. These plants have strong connections to human life because they serve various purposes. Horticultural plants are highly valuable economically. Horticultural plants release volatile compounds with different components and concentrations through various tissues and structures in response to environmental changes to promote the growth and reproduction of plants and enhance their adaptability to the environment [1]. These volatile compounds serve as signals and gradually form modes of interaction between plants, animals, and microorganisms [1]. It is precisely because of these volatiles that horticultural plants possess rich floral fragrances and enticing flavors in fruit, which adds value for their utilization and development in palatable, medicinal, and other aspects [2, 3].

Volatile organic compounds (VOCs) are molecules that are light weight and low polarity with high vapor pressures under normal ambient conditions. These molecules are derived from various closely related biosynthetic pathways associated with general metabolic pathways or products [4]. Plant volatiles can be categorized as floral volatiles, fruity volatiles, or external stress-induced volatiles. They encompass a wide range of compounds, such as terpenoids, phenylpropanoids, derivatives of fatty acids and amino acids, aldehydes and ketones, phenolics and alkaloids [2, 5]. With their complexity and diversity, plant volatiles play significant physiological, signaling, and defensive roles in plant tissues and provide detailed information about plant physiology and ecological status [6, 7].

The silent and colorless micro-volatiles released by horticultural plants can significantly influence the physiological and morphological characteristics of plants, thereby regulating their growth and adaptability [8]. These volatiles can act as attractants to lure pollinators or as repellents to deter herbivores. They can serve as natural and environmentally friendly solutions to enhance plant defense and production by effectively inhibiting plant pests and diseases [1, 9, 10]. Furthermore, they can function as signals to transmit information from the plants to the outside world, thereby protecting and inducing plants to resist biotic and abiotic stresses. This enables them to promote their reproduction, enhance the flavor of their fruits and flowers, and strengthen their ecological adaptability [1, 9, 10]. Researchers also selectively breed high-yielding varieties that are highly resistant and adaptable to consumer preferences based on the patterns of release and functional characteristics of their volatiles to enhance their economic and developmental values [11, 12].

Currently, the study of plant volatiles, particularly terpenoids, has garnered significant attention owing to their compositional analysis, anabolic pathways, regulatory genes, and ecological functions. The analysis of metabolome/transcriptome analysis, genome sequencing, and gene editing technology in horticultural plants, such as lavender (*Lavandula*), *Camellia*, *Solanum*, *Rosa*, and the Orchidaceae, have provided new insights on the importance of volatiles in various applications, including food freshness, spices, essential oils, and pharmaceuticals [12–16]. In addition, the study of signaling pathways involved in the biosynthesis and release of volatiles in horticultural plants faced with stressors, such as herbivores, pathogens, drought, and low temperature, is also a current focus [5, 8]. However, for a comprehensive understanding of the specific mechanisms and ecological functions of volatiles, it is crucial to study not only the biosynthetic and metabolic pathways of volatiles but also the structure and transport pathways of the glands that release them.

Currently, there is a lack of detailed introductory analyses and systematic reviews on the structure and function of the glands that release the volatiles. Few researchers have combined the release of volatiles with the characteristics of horticultural plants to summarize and elaborate on the functions of volatiles in horticultural plants and the specific characteristics of their release. We need to summarize examples of the sites in horticultural plants that release fragrances and the transport and mechanisms of release of these volatiles to illustrate the diversity and functional specificity of these sites. This would further enhance the current understanding of the importance of the release of volatiles for horticultural plants. Studies on the site of the release of volatiles and their mechanism of transport can enable the identification and measurement of specific volatiles, thus, determining the relevant regulatory genes. Relevant studies are also closely related to immunity, mechanism of flower pollination, pest control and other aspects [17, 18]. Currently, the special structure that releases fragrance and the pathways for the release of volatiles of the model plant *Arabidopsis thaliana* and the model plant for floral scent research, *Petunia*, have been extensively studied, thus, laying the foundation for further in-depth exploration of their mechanisms of transport [7, 17, 19–23].

Therefore, this review aims to examine the characteristics and functions of the structures that release volatiles in various horticultural plants and proposes a model for transport to visualize the transport pathways of volatiles that are released from different structures, thus, providing insights into the mechanism of the release of these compounds. Finally, we summarize the patterns of the release of volatiles under the influence of different factors and provide concluding remarks and suggestions for future research based on their ecological functions.

### The sites of release and functions of volatile compounds

Horticultural plants, such as fruit trees, vegetables, and flowers, attract pollinators, deter herbivores, and defend against microbial pathogens by releasing volatiles [2]. Owing to their special structures for the release of volatiles, horticultural plants can fully exert the ecological functions of volatiles, thereby enhancing their economic value in terms of yield, ornamental value, flavor, and other aspects; this enables them to exhibit characteristics, such as prominent aroma traits and strong adaptability [2, 24]. Some horticultural plants are cultivated for their aromatic characteristics or insect resistance, and they possess special structures to release fragrances that can emit unique volatiles [25, 26]. These special structures, which are often composed of epidermal cells or formed through the specialization of epidermal cells, are important sites for the biosynthesis and release of plant volatiles [23, 27].

### Structures that release fragrances

#### Osmophores

Osmophores are the glands in plant tissues that can produce and release volatiles. They typically consist of uniform cell layers, such as ordinary epidermal cells and subcutaneous secretory cells, with specific morphological characteristics that are located in specific parts of plant tissues [28, 29]. Although osmophores, as structures that release fragrant compounds, have been reported in plant organs, such as leaves, they are more commonly found in floral organs [29]. Therefore, osmophores are often described as novel floral secretory structures that secrete scents to attract pollinators and are less frequently mentioned in the context of structures that release fragrances in other types of plant tissues [30].

Osmophores are usually formed by the epidermal cells to the near-axial direction of specialization and development, and they are found in various shapes [28, 29]. These specialized glandular tissue cells, which produce volatiles, have a large nucleus, dense cytoplasm, proteins, and many starch granules and lipid inclusions. These secretary cells have a layer with a strigillated or glossy endoplasmic reticulum (ER), a small number of Golgi complexes, and many mitochondria and liposomes [28, 29]. The surface of these cells is usually covered by a waxy cuticle [21]. The floral organs of most horticultural plants possess osmophores, and they are primarily located on the adaxial surface of the petals. The osmophores of genera, such as rose (*Rosa*) and the Orchidaceae, Boraginaceae, and legumes, share similarities in morphology; they all appear as conical or papillary protrusions (Table 1) [71, 72, 75, 79, 82]. In contrast, the cells of the near-axial surface of the petals are folded in tuberose (*Polianthes tuberosa*), blister-shaped in four-o-clock (*Mirabilis jalapa*), and brush-like in *Osmanthus* [28, 41, 46, 73], and they contribute to the prominent aromatic traits of most horticultural plants. Compared to the flat cell surfaces, the raised or hairy specialized cell structures that protrude or are hairy are more conducive to the release of volatile compounds and also more effective at attracting pollinators and defenders, thereby enhancing the ability of plants to adapt to their environment [73, 83].

**Table 1:** Release sites and characteristics of volatiles from horticultural plants

**Plant species**	**Structure that releases fragrance**	**Structural characteristic**	**Plant organ**	**References**
		PGTs (present in storage cavities), CGTs	Leaves, stem	[31–33]
*Lavandula*	GTs	PGTs	Flowers	
		CGTs	Sepals	
*Salvia*	GTs	PGTs, CGTs	Sepals	[34]
*Perilla frutescens*	GTs	PGTs	Leaves, stem	[35]
*Cannabis sativa*	GTs	Sessile, non-sessile, and bulbous secretory trichomes	Flower petals, bracts	[36, 37]
*Artemisia annua*	GTs	Biseriate trichomes (presence of storage cavities)	Leaves	[32, 38, 39]
*Linaria vulgaris*	GTs	Fascicled trichomes	Flowers	[40]
*Osmanthus*	Osmophores	Presence of MG in the cell and brush-like hairs on the cell surface	Flower petals	[41–43]
*Jasminum sambac*	Osmophores	Presence of MG in the cell	Flower petals	[44]
*Murraya paniculata*	Osmophores and stomata	Cuticular ridges present on petal surface, secretory cuticle present on stamen, supra-stylar head	Flower petals, stamens, stigma	[45]
*Polianthes tuberosa*	Osmophores and stomata	Flat epidermal cells, cuticular ridges (presence of lipid droplets)	Flower petals	[46, 47]
*Dianthus*	Epidermal cells	Cuticle, flaky and tubular epidermal wax	Leaves	[48, 49]
*Camellia sinensis*	Epidermal cells	Cuticle components regulate the synthesis and release of floral volatiles	Leaves	[50–52]
*Ziziphus jujuba*	Epidermal cells	Cuticle wax surface smooth, circular cracks, ridges present at epidermal margins	Fruits	[53]
*Hibiscus trionum*	Osmophores	The epidermal cells are the papillose and cuticle	Flower petals	[54]
*Nymphaea hybrid*	Osmophores	Wrinkled and papillose petals’ surface, and the epidermal cells contain MG	Flower petals	[55]
*Malus pumila*	Epidermal cells	Slabs of cuticle wax cover the fruit to form a film	Fruits	[56]
*Prunus mume*	Osmophores	Protrude outward epidermal cells, and the surface are characterized by curly stripes	Flower petals	[57]
*Zingiber officinale*	Secretory cavities	There are oil cells in the rhizomes	Rhizomes	[58]
*Chrysanthemum*	GTs	GTs capable of storing volatile terpene compounds	Leaves	[59]
	Epidermal cells	Large numbers of stomata, GTs, secretory cavities, osmophores	Floral organs	[25, 26, 60]
*Ribes*	GTs	PGTs, CGTs	Leaves	[61]
*Solanum*	GTs	Storage cavities of type VI trichomes	Leaves	[10, 32, 62]
	Epidermal cells	Cuticle	Fruits	
*Petunia*	Osmophores	Cuticle thickness, component regulation of floral volatile release	Flower petals	[22, 63]
*Michelia*	Osmophores	Presence of MG in the cell	Flower petals	[64, 65]
*Aristolochia*	GTs	Hooked trichomes present on perianth segments, tubular secretory cone trichomes present in tube, filamentous multicellular trichomes on utricles	Flowers	[66]
*Verbena*	GTs	Type IV, V, VI, and VII GTs	Flowers	[67]
*Rosa*	Osmophores	Papillate epidermal cells, presence of cuticle streaks	Flowers	[16, 67–70]
	GTs	Secretion of lipids and terpenes by GTs through cuticle rupture	Leaf blades, stipules, petioles, bracts, calyx, corolla, stems	
Orchidaceae	Osmophores	Conical epidermal cells (osmophores), cracked cuticle releases fragrance	Flower petals	[71–74]
	GTs	Presence of two parallel oval unicellular trichomes on dorsal surface of labellum		
Bignonieae	Osmophores, GTs	Presence of MG in conical and papillary epidermal cells, folded cuticle	Flower petals	[75]
Rutaceae	Secretory cavities	Presence of oil gland in fruit exocarp	Leaves, fruits, flower petals	[45, 76–78]
	Osmophores, stomata	Transparent osmophores, cuticular ridges	Flower petals	
Fabaceae	Epidermal cell	Osmophore, secretory cavities	Flowers, leaves	[76, 79, 80]
Myrtacea	Secretory cavities	Oil glands (presence of storage structures)	Leaves	[81]

A summary of the structures that release fragrances and are found in various plant materials and tissues, including epidermal cells, osmophores, glandular trichomes, secretory cavities, intracellular granular material (matrix granules), and stomata.

GTs, glandular trichomes; PGTs, peltate glandular trichomes; CGTs, capitate glandular trichomes; MG, matrix granules

#### Glandular trichomes

Trichomes are specialized products of epidermal cells with different structures and functions. They can biosynthesize secondary metabolites and also be used as a barrier between plants and the external environment, which provides protection from biotic and abiotic stresses [27, 31]. Trichomes can be categorized into two groups, including glandular trichomes (GTs) and non-glandular trichomes (NGTs), based on their ability to secrete chemicals [27, 31].

GTs are multicellular epidermal structures formed by the specialization of epidermal cells that can conduct biosynthesis and secrete secondary metabolites [24]. GTs are hailed as ‘phytochemical factories,’ which can biosynthesize, store, and release many volatile compounds, as well as secrete other bioactive metabolites [35, 83].

There are two main types of GTs, including peltate glandular trichomes (PGTs) and capitate glandular trichomes (CGTs) [31]. PGTs primarily release volatile compounds and are mostly located on the surface of leaves and sepals. They consist of basal cells, stalked cells, and larger head cells that can secrete metabolites, which are often covered by the cuticle [24, 31, 32]. CGTs primarily secrete sticky resinous substances and are mostly located in the petals and consist of basal cells, longer stalk cells, and smaller head secretory cells with a storage cavity to accumulate metabolites [24, 32, 36]. GTs are found abundantly on the surface of tissues of the Solanaceae, Labiatae and Asteraceae (Table 1) [32]. Many Labiatae plants, such as English lavender (*Lavandula angustifolia*), East Asian sage (*Salvia japonica*), and Korean perilla (*Perilla frutescens*), have both peltate and capitate glandular trichomes [31, 34, 35], and the surfaces of sepals and stipules of rose are primarily covered with capitate glandular trichomes (Table 1) [16]. The morphology and categorization of trichomes can vary in different plant species [24].

PGTs and CGTs all contain storage cavities. This unique cavity structure is formed by the head cells of the GT and promotes the biosynthesis, storage, and release of volatiles; it biosynthesizes and stores high concentrations of volatiles, while limiting their release to the atmosphere; thus, it prevents these high concentrations from re-entering the secretory cells and damaging them [32]. There are three types of storage cavities based on morphology, including shield-like trichomes in lavender, biseriate trichomes in *Artemisia annua*, and type VI PGTs in tomato (Table 1) [10, 24, 32].

The volatiles released by glandular trichomes, which primarily consist of terpenes and phenylpropanoids, play important ecological roles in defending against herbivores, resisting pests and diseases, and attracting predators or natural enemies [10, 83, 84]. Simultaneously, these volatiles are considered to be potential ingredients to make perfumes, essential oils, medicines, insecticides, and food additives; thus, they are highly valuable for their development and utilization [10, 83, 84]. Therefore, glandular trichomes are a promising area of research to enhance the economic value of horticultural plants [10, 24]. The five horticultural plants tomato (*Solanum lycopersicum*), cucumber (*Cucumis sativus*), sweet wormwood (*Artemisia annua*), tobacco (*Nicotiana tabacum*), and cotton (*Gossypium hirsutum*), have become key materials to study the morphology and developmental patterns of glandular hairs [24].

NGTs are known as unicellular non-glandular trichomes and are often dendritic in shape and can be found in the leaves, sepals, and stems [31]. Although NGTs do not have secretory functions, they can physically protect plants from UV irradiation, attack by pathogens, excessive transpiration and also aid in seed dispersal [27, 31, 36]. For example, the non-horticultural plant *Arabidopsis thaliana* possesses typical NGTs and serves as a model plant to study single-cell trichomes [20].

#### Secretory cavities

Specialized epidermal cells can develop outward to form GTs or inward to form secretory cavities [76]. Secretory cavities are subepidermal secretory structures that have developed from multi-layered epithelia and can biosynthesize and store large amounts of secondary metabolites, such as terpenes and phenolics. These structures not only protect plants from the toxicity of high concentrations of metabolites but also provide important chemical defenses for plants against biotic and abiotic stresses [76, 80, 81].

Secretory cavities are commonly found in the leaves and fruits of horticultural plants, such as the Rutaceae, Hypericaceae, Myrtaceae, and Leguminosae [76, 77, 80, 81]. In citrus (*Citrus* spp.) plants, they are also known as oil glands, while in plants, such as the Hypericaceae and Chinese ginger (*Zingiber officinale*), they are referred to as oil cells [58, 81]. Significant breakthroughs have been made in the research on the formation, development, and genetic mechanisms of oil glands. The transcription factor LATE MERISTEM IDENTITY 1 (LMI1) serves as a core regulator of oil gland development [76]. The transcription factor DORNROSCHEN-like (DRNL) can promote the transcription of *LMI1* by binding to the GCC box, a *cis-*regulatory motif in the *LMI1* promoter, thereby regulating the formation of epidermal secretory structures and further facilitating oil gland formation [76, 78]. In addition, the *DRNL-LMI* module can activate another key transcription factor, *MYC5*, to coordinately regulate oil gland structure formation and essential oil synthesis [76, 78]. Due to the presence of oil glands, which act as a ‘reservoirs’ for various metabolites, citrus fruits possess a unique and rich aroma, contributing to their high economic value. Molecular studies on oil gland formation also provide insights for the breeding of disease-resistant, stress-tolerant, and flavor-unique citrus varieties [78].

The high concentration of metabolites biosynthesized and stored in secretory cavities is often responsible for the fragrant and pungent nature of horticultural plants. This structure also makes these plants potential sources for the extraction of essential oils and medicinal components and is closely related to the resistance, fruit quality, processing value, and development and potential to utilize horticultural plants [58, 76, 80].

#### Intracellular granular material

Volatiles accumulate in the fragrance glands of plants in the form of granules, and observing the changes in these particulate substances within cells helps to study the mechanism of the release of plant volatiles [55]. Since intracellular granular material has been proven to play a crucial role in the development and formation of flowers, it has primarily been the focus of research related to the release of aromas in floral organs [41, 47, 55]. However, they have rarely been studied in other plant organs, such as leaves and roots.

The particulate matter located in the fragrance glands of the plant organ can be roughly divided into two categories. One is the lysosome, which is surrounded by a clear halo, possibly owing to the release of enzymes from floral substances or lysosomes. The other is the leucoplast, which is composed of multiple small particles. The quantity, size, electron density, and morphology of these particulates show different changes during the biosynthesis of floral compounds and at different stages of the release of flower fragrances. They are the precursors of floral aromas, energy reservoirs, or enzyme systems for the formation of aromas. The precursors are mostly produced in basic thin-walled tissue cells, transported to the epidermal cells by diffusion, and finally released through the floral stomata of the epidermal cells [44, 64, 65].

Owing to differences in the physical properties among different volatiles, not all volatiles can form granular material. For example, β-violet ketone is volatile and easily forms particles that often gather on the surface of the petal on the special release structure, while linalool is volatile and is often released directly from the petal surfaces, forming fewer particles [41–43]. Some of the granular material contains ester and terpene volatiles that can be oxidized by and combine with osmic acid to form black granular material, which is known as osmiophilic matrix granular material (MG) [41]. MG is an aggregate of volatiles and the basis for their release. They are formed in the cytoplasm and then cascade out of the cell wall and release volatiles through structures, such as trichomes, which are abundant in the epidermal cells [41, 55]. MG is present in the epidermal cells of *Osmanthus* petals, and the brush-like hairs present on the surface of the epidermal cells help to facilitate the aggregation of aromatic granules, which is the basis for the release of aromatic compounds from *Osmanthus* [16, 75, 82]. Such intracellular granular material are also precursors for the release of aromas, energy reservoirs, or enzyme systems for aroma formation in Arabian jasmine (*Jasminum sambac*) and *Michelia* [71, 76, 81]. The petals of the water lily (*Nymphaea hybrid*) contain starch and MG during its flowering period. Their wrinkled, papillose osmophores facilitate the release of these volatile compounds, and denser granular material matter results in the release of richer aromatic compounds [55].

### Tissue-specific release of volatiles

**Figure 1:**
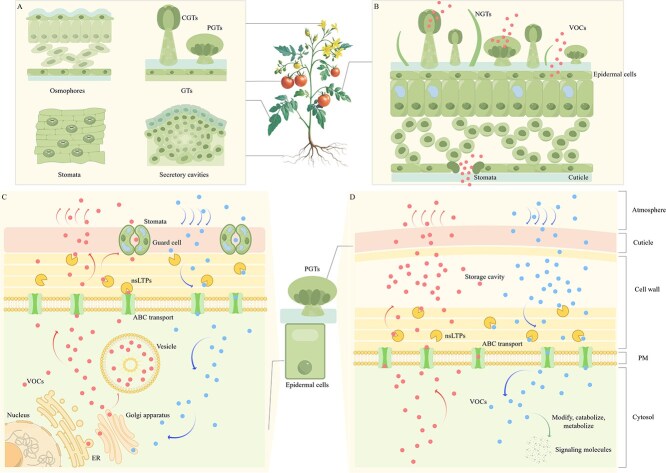
Diagram of volatile release-receiving pattern in horticultural plants. A and B show osmophores, GTs, secretory cavities, stomata, and other important fragrant-releasing structures of horticultural plants are found in different plant tissues such as flowers, leaves, roots, and fruits, releasing volatiles with ecological functions. C illustrates the process of volatile release-reception from the epidermal cells of horticultural plants. Volatiles are synthesized by the nucleus and pass sequentially through the ER, the Golgi apparatus and are transported to the PM by different types of vesicles. The volatiles are then sequentially transported through the PM by ABC transporters, through the cell wall by nsLTPs, and finally into the gas phase by diffusion through the cuticle. Volatiles may also enter the atmosphere directly through stomata after passing through the cell wall. It also demonstrates the process of volatiles being released, sensed and received by plants. D shows the release-reception process of volatiles in GTs with storage cavities (taking the PGTs as an example). The storage cavities can accumulate volatiles at high concentrations to prevent a large amount of volatiles from entering the cells and causing cellular damage. Volatiles that enter the cells will be modified, decomposed, and metabolized to act as signaling molecules. In C and D, the circles on the left represent the volatiles synthesized in the cells, and they form the release path of volatiles with the associated arrows. The circles on the right represent the external volatiles, and they form the path by which the volatiles are sensed and received by the plant, together with the associated arrows. GTs, glandular trichomes; PGTs, peltate glandular trichomes; CGTs, capitate glandular trichomes; NGTs, non-glandular trichomes; VOCs, volatile organic compounds; ABC, ATP-binding cassette; nsLTPs, non-specific lipid transfer proteins; ER, endoplasmic reticulum; PM, plasma membrane.

The biosynthesis of VOCs occurs in almost all the organs of higher plants, but they are primarily released from the vegetative, floral, and root tissues [2, 23]. In the flowers and roots, the VOCs are primarily synthesized in epidermal cells and released into the atmosphere or rhizosphere, while in vegetative organs, such as leaves, the VOCs are often synthesized and released from GTs or directly produced in the mesophyll cells and released through the stomata or cuticle [23]. Of course, plant organs do not rely on a single structure or form to release volatile compounds; they often utilize the diversity of fragrance glands to exert the ecological roles of volatiles and enhance the environmental adaptability of plants (Fig. 1). These VOCs contain complex components, and the production and release of volatiles in plant tissues are usually associated with specific tissues and organs; different tissues synthesize and release volatile metabolites that serve distinct functions [85].

#### The release of floral VOCs

Horticultural plants encompass many varieties with prominent floral fragrance traits, and these floral VOCs are important characteristics of the scents of flowers. Based on the floral sources, biosynthesis, and functions of the VOCs, the floral scent compounds can be classified into terpenoids, phenylpropanoids, fatty acid derivatives, and amino acids [2, 86]. These floral volatiles are primarily released through osmophores, but GTs, which are also structures that release scents, are often present on the surface of floral organs in some plants. Therefore, the components of aromas and the sites of release of the VOCs differ among various types of flowering plants [2].

The osmophores on the petals are the primary fragrant glands in legumes, and they primarily secrete terpenoids, phenolics, and nitrogenous compounds [79]. *Verbena* plants can release scents through both osmophores and GTs on the surface of their tepals [67]. Rose plants primarily utilize osmophores on the upper epidermis of their papillose petals to release lipids, terpenes, and other floral scent compounds, but there are also CGTs on the surface of their sepals and pedicels, which are important sites for the release of volatile compounds [16, 68, 82]. The osmophore cells of Mei (*Prunus mume*) protrude outward and have regular, dense, curly stripes on their surface; they primarily emit aromatic compounds with benzene rings [57]. The GTs on the bracts and flowers of Cannabis are unique structures that synthesize and secrete cannabinoids and can also accumulate monoterpenes and other terpenes [36, 37]. Sclareol is a specialized antifungal metabolite produced by clary sage (*Salvia sclare*a*)*, and it is primarily released from the GTs of the calyx of *Salvia sclarea.* It is primarily synthesized by CGTs [34]. The osmophores of orange jessamine (*Murraya paniculata*) are located in the distal and lateral margins of the petals, anthers and stigmas, and terpenoids and phenylacetones can be released from the petal epidermis, floral stomata, anthers, and stigma cuticle after the flowers have opened [45].

Flower volatiles are key mediators in plant-pollinator interactions, and one of their important functions is to attract pollinators [86]. Specific floral scents attract specific pollinators. For example, benzene volatiles attract moths for pollination; terpenes attract wasps, and methoxy aromatic volatiles attract beetles [85, 86]. Apart from attracting pollinators, floral scent compounds, such as terpenes, also have defensive functions; they repel predators and enhance the resistance of plants to pathogens [87]. For example, linalool can attract insects, such as moths, for pollination. Thus, plants have formed a coevolutionary relationship with insects, while also driving away facultative visitors and inhibiting bacterial growth; they play a crucial role in the proper allocation of resources for reproduction and defense [86, 88]. This mutually beneficial symbiotic relationship between plants and pollinators mediated by scent can significantly impact the yield, resistance, and economic value of horticultural plants [69, 89].

Specialized structures that release scents, such as osmophores and GTs, are also more conducive to visits by pollinators, and the combination of specific volatile compounds and structures that release scents can significantly improve the efficiency of pollination [40, 69, 75]. Specialized petal epidermal cells with conical, papillate, or other special structures can increase the footholds for insects, such as bees, which enhances their likelihood of visitation [75]. For example, the osmophores of *Rosa gigantea* are composed of conical and papillate cells, and they primarily release volatiles, such as homologs of eugenol, that give the plant a unique tea scent, and attract pollinators, such as moths, bees, and fruit flies [69]. Orchids deceive male pollinating insects by releasing female pollinator pheromones composed of multiple compounds through osmophores [72]. The corolla, calyx, and pedicel of common toadflax (*Linaria vulgaris*) have many GTs that attract pollinators or defend against ineffective insect visitors by secreting lipids, phenolics, terpenes, and flavonoids [40]. The flowering organs of Dutchman’s pipe (*Aristolochia*) have a unique morphology and contain trichomes that develop as three different morphologies, including hooked, conical and filamentous, on the tepals and in the floral tube, which have a key role during the process of pollination [66]. The floral scent components of lavender are primarily monoterpenes and sesquiterpenes, which are primarily synthesized and stored in GTs. Among them, linalool, linalyl acetate, and geranyl acetate can serve as attractants for pollinators [31, 33].

Furthermore, the volatiles released by the floral organs of some horticultural plants can serve as both attractants and repellents and attract insects for pollination, while reducing damage to the plants by herbivores [26]. Pyrethrum (*Tanacetum cinerariifolium*) is an ornamental plant, which can resist insects, and primarily releases pyrethrins dominated by (E)-β-farnesene. These chemicals are highly effective insect repellents and are found in flower stalks that are susceptible to infestation with aphids. This plant has a dual defensive function of attracting ladybugs and repelling aphids [25, 60]. Simultaneously, pyrethrum releases a large amount of germacrene D at night, which attracts moths for pollination, primarily through the corolla and stigma where the moths prefer to land [26]. The numerous stomata, GTs, and secretory cavities in the floral organs of pyrethrum facilitate the release of volatiles [26]. Therefore, the plants release specific volatiles from relevant organs at specific flowering stages and times, which match the behavioral activities of beneficial insects [26]. The localization of these special volatiles in floral tissues, the timing of their release, and the flowering habits of the plants collectively promote pollination and resolve the conflicts caused by herbivores [25, 26, 60].

#### The release of leaf VOCs

Green leaf volatiles (GLVs), also known as plant leaf volatiles, are abundant in plants and constitute an important component of the VOCs [90]. GLVs primarily consist of six-carbon chemicals, including alcohols, aldehydes, and esters, which can repel or attract herbivores and their natural enemies, induce plant defenses, or prime plants to enhance their defenses against herbivores and pathogens, as well as exert direct toxic effects on bacteria and fungi [90].

Horticultural plants, represented by vegetables, are highly susceptible to biotic and abiotic stresses, which lead to pest and disease problems that affect their yield and quality [10]. GLVs, which are immediately released upon mechanical damage and biotic stress, serve as instant signals in the plant environment [90]. Studying their biosynthetic and metabolic pathways can provide guidance for the breeding of horticultural plants, target quality formation, and the sustainable development of natural resources and metabolites [9].

GLVs are primarily released through GTs or from the stomata via the leaf mesophyll cells [23]. The GTs on the leaves are crucial for plants to adapt to their environment and overcome biotic and abiotic stresses since they release secondary metabolites that aid in the defense of plants against predators [10, 24, 84]. The numerous VOCs, primarily terpenoids, that are synthesized and secreted by GTs have a role in mediating resistance to insects and fungi, thereby enhancing resistance to infestations and diseases, respectively [83, 91]. Additionally, the presence of NGTs can reduce the damage from UV, water loss, and improve tolerance to freezing, and they work in concert with the GTs to provide both physical and chemical defenses in plants [10, 59].

There are various types of CGTs and PGTs on tomato leaves, which secrete chemicals, such as acyl sugars, terpenoids, flavonoids, and methyl ketones [10]. These chemicals play crucial roles in the resistance to various stresses. For example, acyl sugars have direct defensive effects and protect plants from pathogenic fungi and specialized herbivores; terpenoids repel herbivores or are toxic to them while attracting predators and parasitic natural enemies, and flavonoids have properties that reduce the oxidative damage caused by short-wave solar radiation. Further research is merited to explore the morphology, composition, and functions of different trichomes, which can provide important insights into the elucidation of the mechanisms of plant defense [10]. The surface of *Chrysanthemum morifolium* leaves contains GTs that store and release volatile terpenoid compounds. It has been discovered that the CmMYC2-CmMYBML1 module can prevent feeding by herbivores, such as tobacco cutworm (*Spodoptera litura*) larvae, by regulating the density of trichomes and the content of the biosynthesis of their terpenes [59]. Bamboo (*Bambusa emeiensis*), a food that is rich in nutrients, easily attracts herbivores. Its surface trichomes release volatile compounds to defend against herbivores, while its NGTs provide physical defense functions [92].

The volatiles produced by leaf GTs also have significant industrial and pharmaceutical value. These specialized metabolites can be extracted and formulated into commercial products that range from insecticides and perfumes to food additives and pharmaceuticals, with properties, such as anti-parasitic, antioxidant, antimicrobial, antiviral, and antithrombotic effects [24, 35]. For example, tea (*Camellia sinensis*) leaves are rich in volatile phenylpropanoids/benzenoids, which play a crucial role in the quality of the aroma of tea. Their biosynthesis also responds to mechanisms of abiotic stress, which provides a reference for the physiological functions of plant volatile metabolites [85]. Trichomes are one of the key features involved in the evaluation of tea quality and tea germplasm resources, which indicate high quality [50]. Trichomes are primarily found on the underside of the young leaves and contain high levels of aromatic compounds, such as phytol, acetate, 3,5-dimethylbenzaldehyde, benzyl alcohol, and 2-hexenal, which differ from the quality compounds of tea found in the leaf tissues. The genes in tea that have been identified as specific for trichomes suggest that these trichomes are closely related to the environmental adaptation of tea plants [50].

In addition, perillaketone is the primary metabolite in the leaves of Korean perilla, and the PGTs on the leaves are the primary glands that release this type of fragrance [35]. Lavender releases volatiles from the stems, leaves, and flowers, which all contain different types of trichomes [31, 33]. The GTs of currant (*Ribes*) are primarily found on the upper epidermis of the leaves, including CGTs and PGTs. The primary volatile compounds secreted are 1-octen-3-ol, hexadecanoic acid, and (Z)-3-hexenal, which can be applied in the extraction of essential oils and drugs, as well as food flavorings [61]. Moreover, the different types of storage cavities within the GTs on the leaf surfaces of *Artemisia annua*, lavender, and tomato enable the quick biosynthesis, accumulation and release of high concentration of volatiles [32, 38].

#### The release of root VOCs

Root volatiles encompass a wide range of chemical classes, including fatty acid derivatives, phenolics, benzenoids, terpenoids, alkaloids, and compounds that contain sulfur (S). These volatiles are primarily released from root hairs, which are specialized extensions of root epidermal cells that cover most of the root surface [93]. Root volatiles play a crucial role in regulating plant–plant, plant-insect, and plant-microbe interactions [94, 95]. These interactions are of significant value in enhancing the resistance of plants to biotic and abiotic stresses, which ultimately contribute to an increase in crop yields [93, 96].

The VOCs released by plant roots, which are highly diffuse, can easily spread long distances through soil pores and play crucial roles in biological processes, such as the development of plants, growth of microorganisms, and plant-microbe interactions. These effects include both beneficial and detrimental interactions [93, 94, 96]. For example, garlic or potato-onion plants can influence specific bacteria in the tomato rhizosphere by releasing varying amounts of volatile diallyl disulfide that contains S through their roots, thereby stimulating or inhibiting the growth of tomato plants [97, 98]. The roots of spotted knapweed (*Centaurea stoebe*) release large amounts of sesquiterpenes, primarily (E)-β-caryophyllene, which can significantly enhance the germination of seeds and the individual growth of some neighboring plants in the absence of insect herbivory on those plants [94]. However, in the presence of insect herbivory on neighboring plants, the release of these compounds significantly promotes damage to these plants owing to feeding by insects, which instead inhibits their growth [94]. Therefore, the volatiles released by roots can also reduce biodiversity by altering the interactions between neighboring plants and herbivores, which may represent a form of plant offense [94]. Additionally, the VOCs released by roots can inhibit the growth of pathogens, attract beneficial microbial populations, or alter the patterns of migration of bacteria to benefit the growth of roots [1, 93]. The interactions of root volatiles have been studied extensively in *Arabidopsis thaliana*. Therefore, increasing the amount of research on horticultural plants can enhance the resistance of crops and high levels of their production, while maintaining the ecological balance and environmental stability [96].

#### The release of fruit VOCs

The volatiles emitted by fruits can attract frugivores to disperse their seeds and also possess antibacterial properties [2, 99]. However, their most important function in horticulture is to attract humans. Currently, horticultural breeding is often used to selectively cultivate the aroma of fruits to enhance their edible quality and economic value [2].

The aromas released by fruits are composed of various chemical compounds, including aldehydes, alcohols, ketones, esters, lactones, and terpenes [2]. There are significant differences in the components of aromas among different species, within the same species, and among different varieties [3]. Approximately 30 volatile compounds are considered to contribute to the flavor of tomatoes, and several important aroma volatiles in tomato, such as 2-phenylethanol, 1-nitro-2-phenylethane, and benzyl cyanide, have fruity characteristics and are considered ideal flavors that align with the preferences of consumers [100]. 2-Ethyl-1-hexanol is the primary active aromatic compound in jujube fruits, which have a better overall quality of flavor at half-red maturity [101]. More than 285 different volatile compounds have been identified in mango (*Mangifera indica*) fruits, with the compounds that determine their characteristic fruit aroma typically present in small amounts, including monoterpenes, sesquiterpenes, and volatile oxygenated compounds. The biosynthesis of these volatiles is influenced by the growth and ripening process of the mangoes [102]. Esters, such as ethyl butanoate and methyl butanoate, which have a sweet fruity aroma, are characteristic volatile aromatic compounds of ripe kiwifruit (*Actinidia chinensis*) [103]. The volatiles of wild blueberry (*Vaccinium* spp.) fruits contain a relatively large number of esters, while cultivated blueberries contain a significant amount of aldehydes [3, 104]. The levels of linalool and 2-ethyl-1-hexanol are also the key to distinguish different varieties of blueberries [3, 104]. Cultivated strawberry (*Fragaria* × *ananassa*) fruits release linalool and nerolidol, which emit sweet aromas, such as those of citrus, rose, and apple (*Malus domestica*), while wild strawberry fruits release α-pinene and β-myrcene with unpleasant odors and eugenol with a spicy aroma that attracts insects [105]. The differences in the characteristic aromas between the cultivated and wild strawberries also reflect the approach of breeding by introducing valuable volatiles into modern cultivated varieties based on genetic foundations [105].

Volatiles are typically released through the epidermal cells of the fruit, and structures, such as GTs and secretory cavities, on the surface of fruit also contribute to the formation of aroma traits in fruit. Various types of trichomes exist on the surface of cucumber fruits. GTs are associated with the production of mineral elements, while NGTs are referred to as fruit spines. These trichomes on the fruit surface are important quality traits of cucumbers [10]. Storage structures exist on the surfaces of some plant fruits, such as oil glands that can store volatile substances in the exocarp of fruits of the Rutaceae, including citrus and lemon (*Citrus lemon*) [76, 77, 81]. Oil glands are rich in metabolites such as terpenes, polyphenols, and flavonoids, serving as the sites for the synthesis and storage of essential oils. They are closely related to the quality, resistance, and deep-processing value of economically fruits, and possess significant development potential [76, 78].

In recent years, exploring the regulatory mechanisms of the formation of aromas has become a hot area of research in the fruit industry. Fruit aroma, as an important indicator to evaluate the quality of fruit, necessitates in-depth research into the factors that influence its formation. This provides a theoretical basis for the precise control of the quality of fruit aroma and the improvement of varieties, thereby aligning them with consumer preferences and enhancing their economic benefits [3].

In summary, VOCs are released into the atmosphere from leaves, flowers, and fruits and into the soil from roots. Different types of plant tissues release VOCs with varying components and functions through structurally diverse fragrant glands. They play crucial roles in attracting pollinators, resisting pests and diseases, and enabling signaling between plants and animals. This significantly enhances the ecological adaptability of plants [106]. Highly valuable horticultural plants can leverage the physicochemical properties and aroma characteristics of different VOCs. Based on fundamental research on their biosynthetic pathways, regulatory mechanisms, and genetic traits, horticultural varieties with higher ornamental and developmental value, as well as greater appeal to consumers, can be selectively cultivated [11, 107]. Furthermore, horticultural plants often face biotic and abiotic stresses during their growth and development. It is imperative to further study the transport and mechanisms of the release of VOCs and their interactions with the environment to obtain cultivated varieties that are highly resistant and have high yields and good quality [26]. This also opens up new avenues to utilize the natural biological defense mechanisms between the plants in agricultural applications, which highlights the significant potential of horticultural plants in the field of pest management [25, 60].

### Intercellular transport of volatiles: The process of the emission of volatiles

VOCs have important roles in ecology and are primarily responsible for attracting pollinators, attracting seed dispersers, defending against herbivores and pathogen attacks, responding to biotic/abiotic stresses, and transmitting signals [106]. The VOCs derived from plants must cross many biological interfaces and be translocated from the intracellular biosynthetic sites to the atmosphere before they can participate in interactions in the ecosystem [17]. Significant progress has been made in the research on the biosynthesis of plant volatiles, but the mechanisms of their release from cells merit further study. The study of the mechanisms of the releasee of volatiles in horticultural plants, represented by petunia, is at the forefront of this field [23, 108]. VOCs are usually synthesized in the epidermal cells closest to the atmosphere, and the volatiles need to pass through the cytoplasm, plasma membrane (PM), cell wall, cuticle, or stomata to reach the atmosphere, and secretory trichomes on the cell surface can also synthesize, store, and release VOCs [106, 108–110]. The process of release requires the help of transporters to conduct active transportation rather than relying solely on diffusion, and every interface and barrier in the transport process has a complex regulatory role in the release of VOCs (Fig. 1) [23, 108, 111]. Therefore, the dynamic release process of how VOCs traverse these barriers in intact cells to reach the external environment is a question that merits further study.

### Transport of volatiles across the plasma membrane

The transport of VOCs from the biosynthetic site across the cytoplasm to the PM relies on typical active secretory pathway, i.e. it passes sequentially through the ER, Golgi complex, and *trans-*Golgi network and is transported to the PM by different types of vesicles (Fig. 1) [32]. In addition to its intracellular transport by vesicles, VOCs can also reach the PM via a non-vesicular lipophilic pathway, i.e. by direct contact between the ER and the PM [32, 106].

The passage of VOCs across the PM relies on transport across the PM mediated by proteins rather than by diffusion, and transporter proteins at the PM regulate the release of VOCs. An ATP-binding cassette (ABC) transporter, is responsible for the active transport of a variety of molecules across biological membranes, including heavy metals, terpene-based secondary metabolites, and plant hormones [23, 112]. ABC transporters can transport phenylpropanoids, terpenes, and other volatiles from within the membrane to the cell wall, which facilitates the outward transport of the volatiles, while promoting the balance of volatile concentrations inside and outside the membrane. They serve as important tools for the active transport of volatiles across the plasma membrane [23]. Among them, the ATP-binding cassette transporter G (ABCG) subfamily has an inverse structural domain, i.e. a nucleotide-binding domain and a transmembrane domain, which can act as an homodimer in cellular efflux [23, 112]. Different combinations of dimers can transport different chemicals, such as ABCG half-transporter combinations ABCG 11/WBC 11 and ABCG 12/CER 5. These dimers can transport lipids from the epidermis to the cuticle [113]. Some ABCG transporter proteins are promiscuous with multiple partnerships, and some are specific dimers with particular transport functions [23, 112, 113]. For example, ABCG 11 can form a flexible dimerization partnership, whereas ABCG 12 can only form dimers with ABCG 11 in the epidermal cells, thereby transporting lipids [113]. Most studies on the functions of transport by ABC transporters have been conducted in *Arabidopsis thaliana*, while research on their roles in transporting volatiles in horticultural plants is still in its infancy. PhABCG 1 in petunia petals can transport phenylpropanoid analogs outside the PM, while reducing the content of intracellular VOCs and preventing their concentration from becoming too high and disrupting membrane integrity [23]. In rape flowers (*Brassica rapa* var. *oleifera*) leaves, BcABCG 18 is a transporter carrier for β-stilbene [112].

### Transport of volatiles across cell walls

The transport of volatiles through the cell wall also relies on active transport via transporter proteins. Non-specific lipid transfer proteins (nsLTPs) located in the cell wall are small, and the internal hydrophobic cavity is suitable for binding and translocating different lipophilic molecules, such as cuticle waxes [114]. This transfer protein is an important member of the network that releases the VOCs and binds to terpenes, such as benzaldehyde, 2-phenylethanol, linalool, limonene, phenylpropanoid, or wax-lipid components, and facilitates their transport across the cell wall to the cuticle [108, 111, 114]. Liao *et al.* (2023) found that a decrease in the expression of PhnsLTP 3 in petunia petals resulted in a decrease in the amount of VOCs that reached the cuticle and an increase in the amount of VOCs between the PM and the cell wall [108]. To prevent the accumulation of excessive concentrations of VOCs within the PM, plants prevent the disruption of membrane integrity by decreasing the amount of the VOC precursor phenylalanine and thus, its biosynthesis [108]. Thus, nsLTPs are similar to ABC transporters, which can regulate the intracellular concentrations of VOCs in addition to transporting specific molecules (Fig. 1) [108].

### Diffusion of volatiles in the cuticle

The plant cuticle is a lipidic heterogeneous polymer layer that covers the epidermis of aerial plant tissues, such as stems, leaves, floral organs, and fruits. It regulates the permeability of water, protects plants from pathogen attacks, facilitates gas exchange, and provides mechanical support. It serves as a primary barrier against biotic and abiotic stresses, such as drought, UV radiation, temperature fluctuations, pests, and diseases [48, 53, 110, 115]. For volatile compounds in plants to be released into the atmosphere, they must be transported from their biosynthetic sites within the cells to the outermost cuticle layer and then released from the plant surface. Therefore, the cuticle acts as the final barrier for the release of volatiles and covers the surfaces of structures, such as osmophores and GTs, that emit scents and have a significant impact on the release of these volatiles [64].

The cuticle has three main basic structures, including the outer cuticle, inner cuticle, and middle stratum. Its structural unit is cutin, i.e. 3D matrix polymers/very long-chain fatty acids (VLCFA) and their derivatives. Cutin can be synthesized intracellularly and across the entire cuticle region and form epidermal wax in the outer cuticle that eventually coats the outer cell wall of the epidermis [21, 51, 116, 117]. The biosynthesis and deposition of cutin determine the composition, morphology of the cuticle, and the efficiency of the release of volatiles [54]. The deposition of cutin can form crystalline waxes or cuticular ridges, which contain various types of chemicals, such as alkanes, aldehydes, primary and secondary alcohols, ketones, and esters among others. These wax crystals result in a strongly hydrophobic and lipophilic cuticle, which protects the structures that emit scents and effectively store the VOCs, while promoting their release [110, 118].

The excessive deposition of cutin can lead to an overly thick cuticle or the formation of wrinkles on the cell surface. This wrinkled structure is referred to as a nanoridge [118]. Nanoridges are formed by the excessive synthesis of the cuticle, which continuously accumulates on the surface of the underlying cells in rows, thus, forming narrow parallel ridges that are 100 nm to a few microns wide and are known as cutin ridges [118]. Nanoridges are commonly found on the cells of floral organs, such as petals and sepals. Owing to the limited surface area of the cells beneath the cuticle, the cuticle is stacked in rows on the surface of these underlying cells. When cell division stops or the rate of cell expansion slows down, cuticular ridges form, which appear in a wavy pattern from the apex to the base of the plant tissue [118]. The cuticle is not firmly attached to the underlying cell wall but rather covers it like a loose carpet, which enables it to move with the expansion and contraction of the underlying epidermal cells. This plays an important role in the development of plant organs [118]. For example, during the development of flowers, the formation of ridge structures and conical cells may help to reduce the physical adhesion between the petals and other flower organs, which facilitates the opening of the flowers and their release of volatiles [21, 54, 118].

Volatiles reach the cuticle and enter the atmosphere by diffusion (Fig. 1), but this seemingly simple process of diffusion is regulated by a variety of factors. The release of volatiles is characterized by species and organ/tissue diversity among others and primarily influenced by plant development, temporal rhythm regulation, and biotic/abiotic factors, whereas the rate of volatile transport is related to the components of cuticles, thickness and morphological structure in addition to their own physicochemical properties [22]. There are differences in the effects on different species and chemicals [117].

The cuticle has the greatest role in resisting the release of VOCs and can influence the composition, amount, and dynamic release of the overall volatiles by controlling the mass transfer of individual ones. Alternatively, the wax components that comprise the cuticle are among the main factors that affect the release of VOCs, and the spacing and length of the wax crystals can influence the diffusion of VOCs with high molecular weights, whereas the coverage of cuticular waxes can influence the permeability of the cuticle [110]. The epidermal cells on the petals of the orchid *Phalaenopsis aphrodite* are cone-shaped, and a large amount of wax is deposited on the epidermis. The *n*-alkanes and primary alcohols in its cuticular wax are different from those of other plants [73]. The primary constituents of the cuticle of tea leaves are caffeine and myo-inositol. Their high content of wax enhances the release of tea aromas [52]. Inhibition of the biosynthesis of fatty acid monomers and esters in the cutin wax components of petunia changes the structure of the cuticles, which inhibits the release of low-pressure volatiles, such as phenylethanol and isoeugenol [63]. Cuticular waxes are present both inside and outside of the epidermis of tea leaves, and thicker cuticles result in the biosynthesis of more of the flavor compounds in tea, while the gradual degradation of the cuticular waxes later promotes the release of these aromatic flavors [51, 52]. The primary components of the cuticular wax in jujube fruits are acids and terpenoids, which can enhance the quality of these fruits when stored [53]. They have a smooth cuticular wax surface that has a few ring-shaped cracks, which is presumed to be caused by changes in the biomechanical properties of the pericarp during the process of fruit ripening and senescence and the presence of dense cuticular ridges at the edge of the epidermis [53]. The cuticles of apple fruit primarily include ultra-long chain fatty acid derivatives and triterpenoids, and the content of most of the chemicals decreased and then increased at the node of 90 days after blossoming, while the percentage of coverage by cuticle wax increased continuously as the fruit grew [56].

In addition, the physicochemical properties of the VOCs are also important factors that determine their rate of passage through the cuticle. VOCs with lower molar volumes and molecular weights resist less when passing through the cuticle, thus, the cuticle has size-limiting characteristics for the diffusion of molecules [108, 110]. However, the cuticle is not a barrier to all VOCs; it primarily acts on less volatile compounds, such as isoeugenol, 2-phenylethanol, and benzyl alcohol among others, whereas the release of volatiles, such as methylparaben and benzaldehyde, is primarily driven by biosynthesis [22].

VOCs only pass through the cuticle by diffusion, and their thickness affects the overall release of volatiles [108]. The deposition of smaller cuticles in tuberose facilitates the release of floral volatiles [47]. Liao *et al.* (2021) showed that the petunia ATP-binding cassette transporter protein (PhABCG 12) regulates the thickness of cuticles, which inhibits the biosynthesis of PhABCG 12, and reduces the cuticle thickness, thus, leading to a decrease in the overall release of volatiles that are not released in a rhythmic manner. Alternatively, the *Arabidopsis thaliana* AtABCG 11, 12, and 13 transport wax precursors form inside the cuticle and regulate its thickness [23]. In addition, the contents of intracellular VOCs increase as the cuticle thickness decreases. To prevent the disruption of membrane integrity, inhibitory mechanisms of intracellular feedback are activated to reduce the intracellular contents of volatiles by inhibiting the biosynthesis of phenylpropanoids and ultimately preventing cellular damage [22]. However, the cuticle does not always serve as the main barrier during the release of volatiles. When the cuticle becomes thinner, it is less active in its role of resistance and the resistance to transfer changes and shifts to other barriers, such as the PM and the cell wall, which control the release of VOCs under high biosynthetic fluxes of VOCs [22]. Therefore, the cuticle is not only the main site of VOC storage but also the last barrier for the release of volatiles before they enter the atmosphere, and it can maintain the balance of VOC between inside and outside of the cell, which is an important part of the scent network [17, 22].

### Stomata regulate the release of volatiles

In addition to epidermal cells and cuticles, stomata can also be involved in the release of volatiles (Fig. 1). Volatiles in plant organs, such as petals and leaves, can be released directly into the atmosphere through stomata [45, 47, 119]. In addition, the VOCs synthesized in the leaves can also be released from the cell wall directly into the sub-stomatal air spaces that connect to the stomata and then released to the atmosphere through the stomata where they receive less resistance than if they were released from the cuticle [106]. Researchers can study the involvement of stomata in the release of compounds by localizing the composition of volatiles in the stomata at the different stages of plant development. The presence of many floral stomata on the proximal axial surface of the tuberose tepals is related to their tissue-specific release of floral compounds; they are covered above the epidermal cells rather than embedded in the epidermis and can be involved in the release of terpenes, esters, and other floral compounds [46, 47]. The guard cells in the stomata function in concert with their neighboring epidermal cells to release volatiles, and some stomata collect cuticular wax ridges on their surfaces; the release of volatile lipids into the atmosphere through the cuticle is also associated with these stomata [45, 47].

### Mechanisms of the transport of volatiles in glandular trichomes

Similar to epidermal cells, secretory trichomes have a similar mechanism to transport VOCs where they can be transported from secretory cells in glandular trichomes across the cytoplasm, PM, and cell wall, directed to the storage cavities, and finally to the cuticle for release [32]. The storage cavities of glandular trichomes with special functions play an important role in plant–animal interactions, and the large number of highly concentrated volatiles that accumulate in the storage cavities of glandular trichomes can be rapidly released when the plant receives a stress signal; this provides a mechanism for rapid response that does not require upregulation of the relevant metabolic pathways and efficiently transmits information to neighboring plants (Fig. 1) [1]. Currently, the VOC transport proteins in trichomes have not been identified, but it has been shown that nsLTP can secrete terpenoids from the storage cavities of trichomes; e.g. the LTP in *Artemisia annua* glandular trichomes binds and carries sesquiterpene lactones across the cell membrane, and members of the ABCG subfamily, which is related to transport, are highly expressed in the glandular trichomes [32, 108, 120]. Glandular trichomes have also become a hot spot in the field of biometabolism because of their cavities for long-lasting storage, the controlled release of VOCs, and their unique mechanism for their precise transport.

## Regulation of the release of volatiles

### Influence of internal plant factors on the release of volatiles

The content of synthetically released VOC in plants varies with the degree of development of their tissues/organs, and the role of different VOCs on the growth and development of plant organs varies. The dominant studies have been conducted on floral volatiles.

Large quantities of the volatiles released from flowers are used for perfumery, cosmetics, flavoring, and medicinal purposes, and the release of these volatiles is affected by a complex of factors both inside and outside of the plant that modulate the ecological interactions between plants and animals [121]. The volatiles released from floral organs are often closely related to the development of flowers. Boachon *et al.* (2019) found that petunia terpene synthase 1 (*PhTPS 1*) regulates the biosynthesis and release of terpenes in the enclosed space of flower buds before the petunia flowers open, which promotes the development of floral reproductive organs, such as the pistil and stigma. The gradual development and maturation of the floral organs prompts *PhTPS 3* and *PhTPS 4* to biosynthesize sesquiterpenes that accumulate on the pistil and stigma to protect the reproductive organs from microorganisms after blooming, which provides petunias with an advantage during the evolution of plant reproduction [122]. The development of flowers is also accompanied by morphological changes in the cuticle of the petal epidermis, which affects the release of volatiles. During the process from budding to the opening of flowers, the cuticle thickens, and starch granules and liposomes appear in the petal epidermal cells; this contributes to the release of many floral volatiles that are synthesized at this time, whereas the cuticle thins during the later stages of flower opening, and the cuticular ridges decrease, with a subsequent decrease in the number of volatiles released by the flower [45]. In addition, transcription factors that regulate the biosynthesis of floral aromas are also involved in the process of releasing volatiles. During the early stages of flower development in rose, floral scents are synthesized in small amounts, whereas during the rapid stage of growth of flowers, some genes related to the formation of aromas, such as the orcinol *O*-methyltransferase 2 (*OOMT 2*) gene, are expressed; the biosynthesis of floral scents reaches a peak, and the *OOMT*s become increasingly involved in the movement of membranes to promote the release of the volatiles by the transporter [70].

In addition to floral development, the production of heat by flowers can also influence the biosynthesis and release of floral scents, which plays an important role in pollination. Osmophores can promote the release of floral aromas through a combination of an increase in the surface area of petals and the production of heat by the flower, with osmophores consuming starch and lipids to facilitate the transport of volatiles to the gland. This process is accompanied by the production of heat, which enables the plant to increase the temperature of its flowers through the metabolic production of heat, thereby increasing the rate of volatilization of floral aromas and promoting the release of volatiles, while attracting pollinators and increasing the level of mating, locomotion, and other activities during their visits to the flowers [123]. In addition, the release of glycosylated volatiles, such as geraniol, geranyl acetate, and hexyl acetate, which are stored within the petals, is thought to be regulated by the plant’s biological clock [70].

### Patterns of release under biotic and abiotic influences

**Figure 2:**
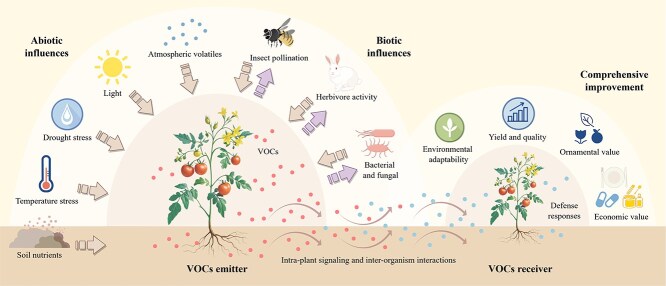
Ecological interactions model of volatiles in horticultural plants. The leaves, flowers, roots, fruits and other parts of plants can all release volatile substances, and the release of these substances is also influenced by various biological and non-biological factors. These include mechanical damage, drought stress, temperature changes, pathogen infestation, herbivore attacks, and so on. Plants, when stressed, also release special volatiles that serve ecological functions such as attracting pollinators, repelling herbivores, and defending against pathogen infections. Additionally, after being stressed, plants also use volatiles as signals to transmit information to neighboring plants, which then initiate defensive responses to cope with the stress after receiving the signals. These volatiles are beneficial for horticultural plants in enhancing their environmental adaptability, improving yield and quality, and increasing ornamental and economic value. The arrows pointing to the VOCs emitter indicate the impact of external factors on horticultural plants, and the arrows pointing to biotic influences represent the effects of volatiles released by horticultural plants on other organisms.

In addition to genetic factors, the variability of the release of volatiles can be influenced by the environment, and numerous studies have shown that biotic/abiotic factors, such as atmospheric volatiles, temperature, moisture, soil nutrients, bacterial, fungal, and herbivore activity, and insect pollination can affect the release of volatiles (Fig. 2) [86].

Abiotic stresses can induce the rhythmic release of VOCs, and most diurnal plant behaviors are synchronized with the daily rhythms of abiotic factors, such as light and temperature. Plants often adjust to these external factors by regulating their own metabolic rhythms to fit the circadian rhythms in the environment to improve their adaptability. This is reflected not only in flower opening and leaf movement but also in the release of plant volatiles; the timing of this release, such as their components, plays an important role in intra-plant signaling and inter-organism interactions [4]. For example, after the leaves of the tea plant are subjected to abiotic stresses, such as mechanical damage or low temperature stress, the plant will produce wound stress responses, thereby promoting the synthesis of indole and (E)-nerolidol synthase [124]. Plants can regulate the rhythm of the release of floral volatiles based on environmental factors, such as temperature, light, and diurnal changes, thereby signaling pollinators. This enables the plants to store energy in a timely manner and effectively release volatiles when the pollinators are active. Simultaneously, it drives away predators and large animals that steal nectar and pollen, which protects the flowers from microbial infection [86, 121]. In addition, the patterns of circadian emission of plant volatiles may also be influenced by the circadian regulation of emission mechanisms, such as the opening of stomata or the transport through membranes [4].

In addition to climatic conditions, environmental factors such as soil status and the plant-associated microbiome also have significant impacts on the synthesis, accumulation, and release of plant secondary metabolites [125, 126]. They are closely related to the formation of plant aroma and fruit flavor [126, 127]. Under soil conditions of high salinity, magnesium, manganese and potassium, *Citrus reticulata* ‘Chachi’ can enhance the content of monoterpenes by up-regulating the expression of salt stress response genes and terpene synthases [125]. Diverse microorganisms in the soil can interact with plants by inducing immune responses or altering metabolic pathways [125, 127]. For example, rhizosphere microorganisms can activate the terpene synthesis pathway or provide precursor substances, thereby enhancing the accumulation of monoterpenes in citrus and fully exploiting their medicinal potential [125]. Microorganisms can also alter the aroma profile of tomato fruits by regulating the synthesis of volatile compounds such as phenylpropanoids and apocarotenoids [126].

Plants biosynthesize and release VOCs in response to biotic stress, which plays an important role in plant defense; they are important mediators of the interactions between plants and their surrounding physical and biological environments and can rapidly release, regulate, and coordinate the adaptive responses of neighboring plants before the onset of competition or potential threats, which can have a significant impact on the ecological interactions between plants and animals [1]. Stress induces the biosynthesis and release of specific VOCs, which can be used as reliable signals for communication between plants and animals; such interactions include herbivore-induced plant volatiles, which are among the most well-studied induced phenomena in the field of plant-herbivore interactions. Herbivore-induced plant volatiles (HIPV) usually include GLVs, terpenoids, phenylpropanoid/benzene, aromatic compounds and amino acid derivatives [4, 90, 128]. HIPV can also be used as a toxin or repellent against herbivores or to attract their natural enemies to prevent them from visiting and laying eggs and promote the production of flowers, buds, and fruits, as well as the transmission of defense signals within the plant [4, 128]. GLVs are released almost immediately by plants when they are stressed by herbivores, thus, they serve as an immediate and informative signal to many organisms in the plant’s environment [90]. GLV is probably the most conserved volatile signal that mediates plant–plant interactions; it triggers a range of defense responses in a wide variety of plants, such as *Arabidopsis*, tomato, and maize (*Zea mays*) [128, 129].

The presence of herbivores can influence the release of volatile compounds by plants, and related research has been conducted extensively on horticultural plants that are both ornamentally and economically highly valuable, such as tea plants. The attack of black pine (*Pinus nigra*) by the pine processionary caterpillar (*Thaumetopoea pityocamp*a) promotes the abundant release of bornyl acetate from the pine needles, particularly during periods when the insects feed intensively. Bornyl acetate is also considered the most common compound closely related to pest infestation [130]. The stress on downy birch (*Betula pubescens*) by winter moth larvae (*Operophtera brumata*) significantly increases the release of terpenes, such as linalool, ocimene, 4,8-dimethylnona-1,3,7-triene, 2-methyl butanenitrile, and benzyl nitrile, as well as sesquiterpenes and GLVs in areas at low altitude [131]. Upon attack by herbivores, such as the tea geometrid larvae, tea plants can induce the biosynthesis of volatile terpenes, such as geraniol and linalool, in the leaves, which, in turn, triggers the release of ocimene by neighboring undamaged tea plants [8, 124]. These terpene compounds have direct defensive functions against insects and serve as typical wound signals that can help to protect the tea plants [8, 124]. In addition to producing stress responses, tea plants can also effectively improve the components of their aroma under biotic stress. For example, attack by the tea green leafhopper can induce the plant to produce and release a unique tea aroma compound known as diendiol I [124].

Plants also release a variety of VOCs to defend against pathogens after infection by pathogens, including terpenes, aromatics, nitrogenous compounds, fatty acid derivatives, and volatile plant hormones [8]. VOCs can enhance the resistance of plants to various pathogens, and plants respond to pathogen infections by activating multi-layered defense systems [8]. For example, tea plants enhance their resistance by releasing salicylic acid, jasmonic acid, and ethylene to activate their system after infection by pathogens [8]. Compared to the extensive research on the interactions between plant volatiles and herbivores, there has been less research on the interactions between volatiles and microorganisms. However, knowledge in this area is increasing, and this interaction can be utilized to reduce diseases in agriculture and forestry [8].

In addition to herbivores and pathogens, pollinators also influence the release of plant volatiles. Many flowering plants require the aid of pollinators to transfer pollen between their flowers; thus, the flowers use volatiles to attract the pollinators by producing specific ones at particular times to synchronize with their activity [4]. Plants depend on pollinators for reproduction, and pollinators depend on pollen and nectar for survival. This mutually beneficial symbiotic relationship produces different floral signals depending on the species. Floral aromas also play an important role in the construction of complex signaling pathways that affect the interactions of plant-pollinator [89]. Volatiles are attractive to pollinators where specific substances attract specific pollinators, such as benzene volatiles that attract moth pollinators, terpenes that attract wasp pollinators, methoxy aromatic volatiles that attract beetle pollinators, and volatile fatty acid derivatives, that are similar to the sex pheromones of insect pollinators, which attract pollinators, as well as the natural enemies of herbivores to visit the plant [132]. The pattern of the release of the floral scent of Japanese honeysuckle (*Lonicera japonica*) represents an ecological adaptive strategy aimed at matching the times that visitors to the flowers are active to enhance the efficiency of pollination and the success of plant reproduction [133]. The rhythmic nature of the release of floral scents may help to increase the efficiency of this process by ensuring that maximum attraction signals are available when the flower visitors are active [133]. Several important horticultural crops, such as blueberries, apples, litchi (*Litchi chinensis*), and cucurbits (*Cucurbita* spp.), rely heavily on volatiles for pollination [107]. Volatiles serve as crucial mediators of communication between plants and pollinators, and further study is needed into aspects of this type of communication, such as the rhythmicity of their release, composition of their chemicals, and ecological significance.

## Concluding remarks and future perspectives

### Research status and the future value of fragrant glands

There is a diverse morphology of the plant glands that release volatiles, and their unique structural characteristics are often observed and recorded. This facilitates the identification of different plant species and the exploration of the development of rules. Rose plants serve as an excellent example of this. For example, beach rose (*Rosa rugosa*) and *R. bracteata* have GTs that can release volatiles and secrete sticky substances on their sepals, peduncles, and leaves, while *R. odorata* var. *gigantea* and other plants have inconspicuous GTs structured. In addition, there are also differences in the morphology of trichomes in different plant species and parts of the same species, including spiny, pubescent, pubescent, and villous [16]. Furthermore, the structure of cuticular ridges is present on the surface of the epidermal cells of the petals of most of the plant material of the Sect. Chinese DC. and the S. Banksianae, whereas the cuticle of the plant material of the S. Laevigatae is smoother. Currently, the structures that release fragrances and the volatile characteristics of most plants in *Rosa* L. have been documented in ‘Genus *Rosa* L. in China,’ thus, providing important clues for the classification and treatment of the genus [16].

In addition to the *Rosa*, the fragrance release structures of *Magnolia*, champac, *Osmanthus*, lilac (*Syringa*), jasmine and other Xylariaceae with outstanding floral aroma traits, as well as *Phalaenopsis* and other orchids, often receive much attention [41, 72, 73]. The structures that release fragrances, while serving as a basis for inter-varietal classification, are often analyzed in conjunction with their characteristics of volatile components. The morphological features of these structures, such as glandular hairs, epidermal cells, and cuticles, also change with the growth and development of plants; there is a close correlation between their patterns of development and the rhythm of the release of volatiles [22, 106]. Therefore, the morphological differences in different structures that release fragrances can provide important evidence to classify plants. Based on morphological, cytological, and molecular biological research, new revisions can be made to different plant groups, which can also have a strong effect on the strategies used for horticultural breeding. Additionally, exploring the developmental patterns of structures that release fragrances can provide references to elucidate the mechanisms used by plants to release fragrances.

However, the current research on the structure of fragrance glands is only at the level of observation by the naked eye or microscopic, and there is little systematic classification and review of the structural characteristics of different GTs. There has also been little in-depth research at the cellular and molecular level on the biosynthesis and release of volatiles and their regulatory mechanisms. Some scientific issues related to the structure of fragrance glands are often overlooked. For example, the development and formation mechanisms of these glands remain underexplored. The regulatory mechanisms controlling the secretion of VOCs from the glands to storage cavity are not well understood. Additionally, little is known about the biosynthesis and transport pathways of cutin precursors. The function of special structures, such as cuticular ridges, in promoting the release of VOCs has not yet been explored. Currently, the metabolomes, genomes, and volatile synthetic metabolic pathways of plants with special aroma traits, such as grape (Kyoho, a hybrid between *Vitis vinifera* and *Vitis labrusca*), lily (*Lilium* ‘Siberia’), thyme (*Thymus vulgaris*), and Biondi’s magnolia (*Magnolia biondii*), have been discovered [134–137]. However, they are seldom used to relate the structures that release volatiles to the aroma traits from the perspective of plant cytology and explore the process of the transport of volatiles and the mechanism of the regulation of the formation of special release structures for volatiles on the plant’s aroma.

Horticultural plants, including flowers, fruits, vegetables, and teas, are well-known for their edible, medicinal, and ornamental value. Numerous studies have been conducted on horticultural plants because of their high economic value. The volatile compounds released by horticultural plants, such as floral and fruit scents, have gradually become a hot area of research. However, to date, no researchers have systematically reviewed the fragrance glands and characteristics of the release of volatiles from horticultural plants or elaborated on the functions of horticultural plant volatiles in conjunction with their economic value. Therefore, we conducted a comprehensive review of the fragrance glands and their functions, characteristics of volatile transport, and factors that influence the release of volatiles in various horticultural plants. This review provides a theoretical basis for the identification and functional determination of various fragrance glands, the further elucidation of the mechanisms of the release of volatiles, and research on the ecological functions and transmission mechanisms of volatiles. More importantly, it offers valuable insights for the breeding of horticultural plants.

### Research progress and prospects of volatile release mechanisms

VOCs have a variety of ecological functions, including the attraction of pollinators, direct defense against herbivores, attraction of the natural enemies of herbivores, and intra- or inter-plant signaling. VOCs can serve as cues between plants and other organisms, including herbivores, pathogens, pollinators, parasites, and plants, which is highly significant in ecology and highly valuable research (Fig. 2) [31, 138].

Plants release VOCs to provide other organisms with information about their physiological and ecological status and also receive external VOC signals to initiate the mechanisms of plant defense [1, 139]. The mechanism used by plants to sense VOCs and transport them into the cell is similar to their mechanism of release. An external VOC first enters the internal space of the plant through the stomata or cuticle. There is little resistance for the stomata to receive a VOC. This makes it easier for the plants to reach a balance between the internal and external concentrations of the VOCs, while the cuticle, which has a larger surface area and is more lipophilic, is more prone to capture transiently transmitted VOCs. The VOCs then reach the cell wall by diffusion. Similar to outward transport, the VOCs may also be transported to the PM by the non-specific lipid transfer protein LTP and then across the membrane by binding to receptors localized on the PM, which can transmit signals of damage to trigger plant immunity, and play a role in protecting the plant from diseases. VOCs can also be translocated across the PM by the LTP or be directly partitioned into plasma or extracellular vesicle membranes, where it enters the plasma membrane by cytophagy. VOCs within the PM may be partitioned into subcellular membranes or bind to compound-specific receptors located in the cytoplasm, ER, or nucleus, which are then modified, catabolized, metabolized, and act as signaling molecules (Fig. 1) [7, 128, 129, 140].

Currently, there are still many issues worthy of in-depth study in the complete regulation of the biosynthesis of plant volatiles and the release, reception, and degradation of the transport process, such as the mechanism of plant sensing and transport of volatile substances, role of external VOC signals on the regulation of the receptor plant, discovery of a variety of transporter proteins, classification, and structure and mode of transport among others. All these issues merit further study and have broad prospects.

Alternatively, these volatiles not only play crucial roles in the survival of plants but also have extensive and significant applications in human life. Horticultural plants are highly relevant to humans. The volatiles they release with their rich and attractive fragrances can be used in the cosmetics industry and as flavorings in the food industry. The medicinal properties of volatiles are also widely applied in the pharmaceutical industry [2, 107]. Therefore, the rational screening of volatile components from horticultural plants and the maximization of the production of volatiles will help to increase the commercial and economic value of these plants (Fig. 2) [107]. Despite advancements in horticultural breeding, it remains challenging to use volatile traits to cultivate high-yielding varieties that are resistant to pests and pathogens, highly ornamental and contain the desired nutritional and flavor characteristics [11, 107]. This process necessitates a combination of various molecular techniques, metabolic data, and biological network models to gain a deeper understanding of the biosynthetic pathways of volatiles and their mechanisms of release. This will provide insights to enhance the aromatic characteristics of horticultural plants and establish a theoretical foundation to cultivate horticultural varieties with high levels of desired natural compounds. Thus, this will ultimately offer tangible benefits to consumers.

In summary, it is highly desirable and of great importance to explore the connection between the structure of release and transport of volatiles, and to analyze the complete transport pathway.

## Data Availability

All the data are presented in the main text file.
